# The detail of the en bloc technique and prognosis of spleen-preserving laparoscopic distal pancreatectomy for pancreatic cancer

**DOI:** 10.1186/s12957-015-0735-y

**Published:** 2015-11-25

**Authors:** Zhipeng Sun, Yubing Zhu, Nengwei Zhang

**Affiliations:** Oncology Surgery Department, Peking University Ninth School of Clinical Medicine (Beijing Shijitan Hospital, Capital Medical University), 334room, Administrative Building, Beijing, China

**Keywords:** Spleen-preserving, Laparoscopic distal pancreatectomy, Pancreatic cancer

## Abstract

**Background:**

Although laparoscopic spleen-preserving distal pancreatectomy surgery is more and more popular, the reports about the en bloc technique used for pancreatic cancer were still rare. The aim of our study was to illustrate the detail of the spleen-preserving en bloc technique as well as the short-term and long-term outcomes.

**Methods:**

The detail of the en bloc technique with pictures was described. The prognosis of the successive 23 cases that underwent the laparoscopic distal pancreatectomy (LDP) surgery was evaluated.

**Results:**

There were 17 cases that underwent spleen-preserving LDP while six cases underwent spleen-resecting LDP. The average surgery time was 203 ± 54 min, and the average blood loss volume was 208 ± 264 ml; one case transferred to open surgery because of severe adhesion. The complication rate was 47 % (*n* = 8) shortly after surgery. Pancreatic fistula rate was 41 % (*n* = 7). No lethal case occurred. The average diameter of the tumor was 32 ± 12 mm. The average number of the lymph nodes obtained was 19.8 ± 9.3. All the cutting edges were negative. Survival rates of the patient after 1, 3, and 5 years are 64.7, 52.9, and 41.2 %, respectively. These records showed no statistical significance compared with spleen-resecting LDP and open distal pancreatectomy (ODP) surgeries.

**Conclusions:**

The en bloc spleen-preserving LDP can be performed by experienced surgeons. This surgery has good short-term and long-term outcomes.

## Background

In the past 20 years, laparoscopic pancreatectomy surgery has been recognized and performed gradually from diagnostic laparoscopic exploration, specimen biopsy to distal pancreatectomy, and pancreatoduodenectomy [1]. Because the procedure of laparoscopic distal pancreatectomy (LDP) was not very complex, the number of cases that underwent this surgery raised quickly for benign and low-grade malignant disease [2].

Although there were many articles about laparoscopic distal pancreatectomy reported [3], the en bloc concept was rarely mentioned. Many procedures reported [4] only contain the distal pancreatectomy technique excluding the ganglion resection, lymph node resection, Gerota fascia removal, and Toldt fascia removal. These procedures were not suitable for pancreatic cancer.

Depending on the Japanese General Rules for the Study of Pancreatic Cancer [5], we designed an en bloc spleen-preserving LDP procedure which removes the Gerota fascia, the Toldt fascia, and the distal pancreas as a whole and resects the 1st and 2nd station lymph nodes (Table 1). Spleen-preserving LDP against pancreatic cancer may be still controversial, and until now, there has not been any report from a single center about the long-term prognosis and the standard en bloc LDP procedure of the surgery. Our aim is to describe the detail of the standard en bloc technique and to evaluate the short-term and long-term prognoses compared with the previous studies [6–10] about open distal pancreatectomy (ODP).

**Table 1:** Pancreatic lymph node group in the General Rules for the Study of Pancreatic Cancer

	Pancreatic tail
1st group	8a, 8p, 10, 11p, 11d, 18
2nd group	7, 9, 14p, 14d, 15
3rd group	5, 6, 12a, 12b, 12p, 13a, 13b, 17a, 17b, 16a2, 16b1

## Methods

From the year 2007 to 2010, there were 60 patients who underwent distal pancreatectomy (DP) surgery. Among them, 45 cases underwent LDP surgery, 15 cases underwent ODP surgery, and 23 cases had pancreatic cancer in the LDP surgery group. Before the year 2002, the indications of LDP were benign diseases. After 2002, LDP was indicated both in benign and malignant diseases. All the patients’ medical records and pathology results were reviewed.

### Ethics, consents, and permissions

The demerits of LDP against pancreatic cancer were the high requirements of surgical skills and the uncertain outcomes. The merits of LDP were the micro-invasive effects leading to fast recovery.

This retrospective study was approved by the Peking University Ninth School of Clinical Medicine ethics committee. Patient records were de-identified prior to analysis. All the patients signed informed consents before surgery.

### The surgical procedure of en bloc LDP

We have three principles in the concept of en bloc spleen-preserving LDP surgery. First is to dissect from the Gerota fascia to the Toldt fascia exposing the left kidney, left renal vein, left adrenal vessels, and left adrenal gland. The en bloc technique dissects the Gerota fascia, Toldt fascia, and distal pancreas. Second is to dissect the lymph nodes and ganglions along the celiac trunks while resecting the lymph nodes and ganglions along the superior mesentery artery (SMA). Third is, if the spleen and the left gastro-epiploic vessels were not invaded, to dissect the spleen vessels but preserve the spleen.

**Fig. 1:**
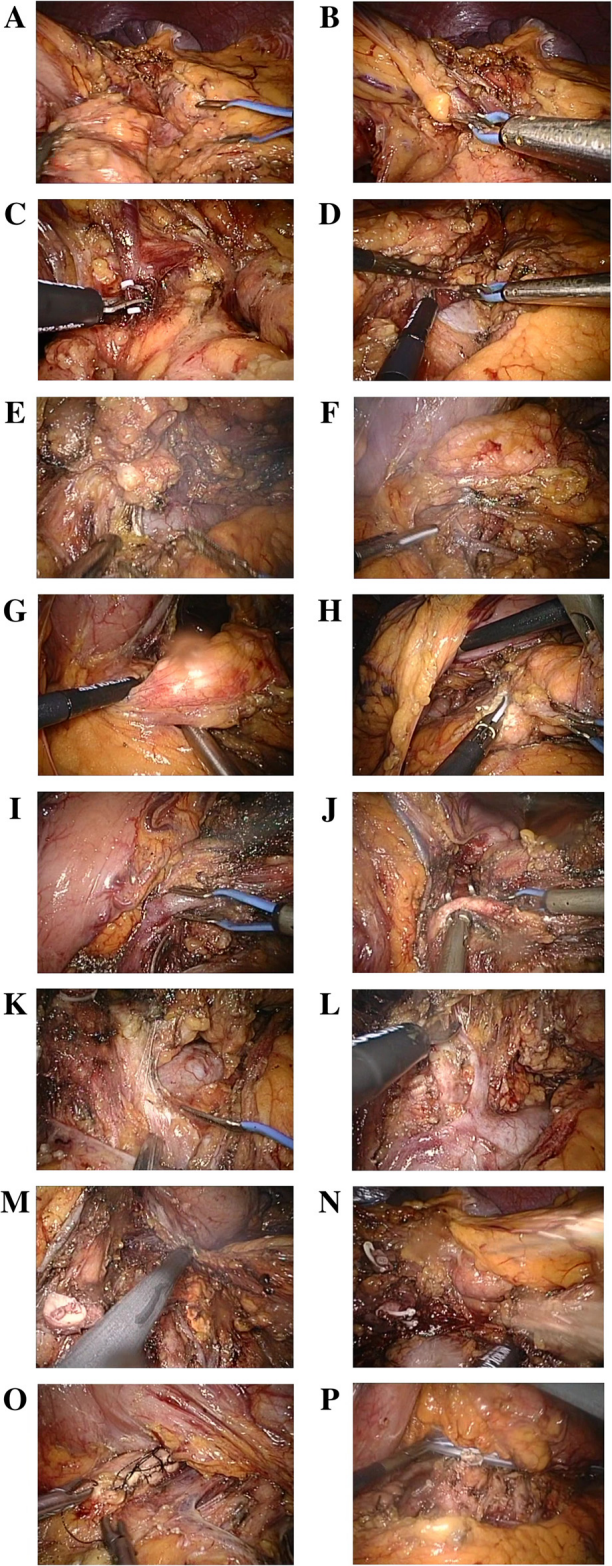
The technique of standard en bloc spleen-preserving LDP. **a** Dissect the gastro-colic ligament and disconnect the transverse mesocolon from the Gerota fascia. **b** At the level above the upper surface of the pancreas, isolate the left gastro-epiploic vein (LGEV) and the communicating veins from the distal pancreas. Preserve the LGEV. **c** Dissect between the communicating vessels and the distal pancreas. **d** At the tail of the pancreas, dissect the Gerota fascia from the lower border of the left renal vein upward to the left kidney. **e** Continue to dissect the Gerota fascia to the left border of the superior mesentery artery (SMA). Dissect the lymph nodes and celiac ganglion group II around the SMA preserving the 5-mm ganglion at the right side. **f** Continue to dissect upward to the splenic vein. Expose the splenic vein and IMV and ligate and cut off the IMV. **g** Penetrate the pancreas from the posterior surface at the root of the splenic vessels. **h** Dissect the pancreas at the root of the splenic vessels with a Harmonic scalpel. **i** Isolate the splenic vein at the root and dissect it. **j** Isolate the splenic artery at the root and dissect it. **k** Dissect the lymph nodes and celiac ganglion group I around the celiac trunk preserving the 5-mm ganglion at the right side. **l** Dissect the retroperitoneum adipose tissue at the upper border of the pancreas. Expose the left adrenal vessels and adrenal gland. **m** Preserve the adrenal gland if it has not been invaded. Dissect the retroperitoneum adipose tissue to the origin. **n** Ligate and dissect the spleen vessels at the tail of the pancreas. **o** Invertedly suture the stump of the pancreas. **p** Leave a drainage tube in the surgical site

The detail of the technique is depicted as follows (Fig. 1).

The patient was placed in a lithotomic position. We inserted 10-mm trocars in the umbilicus and right mid-clavicle line below the costal margin separately, and double 5-mm trocars in the left upper quadrant of the abdomen. After dissecting the greater omentum, we divided the mesocolon transversum from the Gerota fascia. Then, we identified the inferior border of the pancreas.

We dissected from the Gerota fascia to the Toldt fascia exposing the left kidney, left renal vein, left adrenal gland, and inferior mesenteric vein (IMV) with the en bloc method from lateral to medial. We retracted the pancreas from the dorsal side to the ventral side. We exposed the junction of the spleen vein and superior mesentery vein (SMV) from the dorsal side of the pancreas. We mobilized the SMA and resected the lymph nodes and celiac ganglion group II at the left side of the SMA.

We dissected the parenchyma of the pancreas at the root of the spleen vein. After that, we ligated the spleen vein and spleen artery at the root. We continued to resect the lymph nodes and celiac ganglion group II at the left side of the SMA. We preserved the right half of the celiac ganglion group II at this place. We dissected the adipose tissue upward to the cephalic side. We exposed the celiac trunk and resected the lymph nodes and celiac ganglion group I at root of the celiac trunk. We preserved the right half of the celiac ganglion I. Then, we moved to the left. We skeletonized the left adrenal gland and resected the adipose tissue and lymph nodes around it.

Finally, at the tail of the pancreas, we ligated and dissected the spleen vessels and preserved the gastro-epiploic vessels and short gastric vessels.

**Fig. 2:**
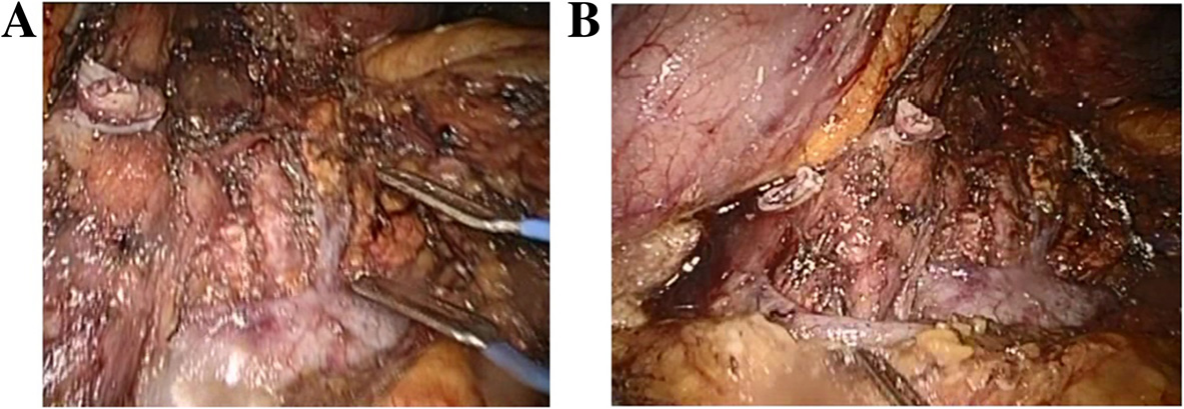
The posterior peritoneal structure after dislodging the specimen. **a**. The stump of the spleen artery, left renal vein, left adrenal vessels, and SMA was shown in the picture after surgery. **b** The stump of the spleen vein was shown in the picture

**Fig. 3:**
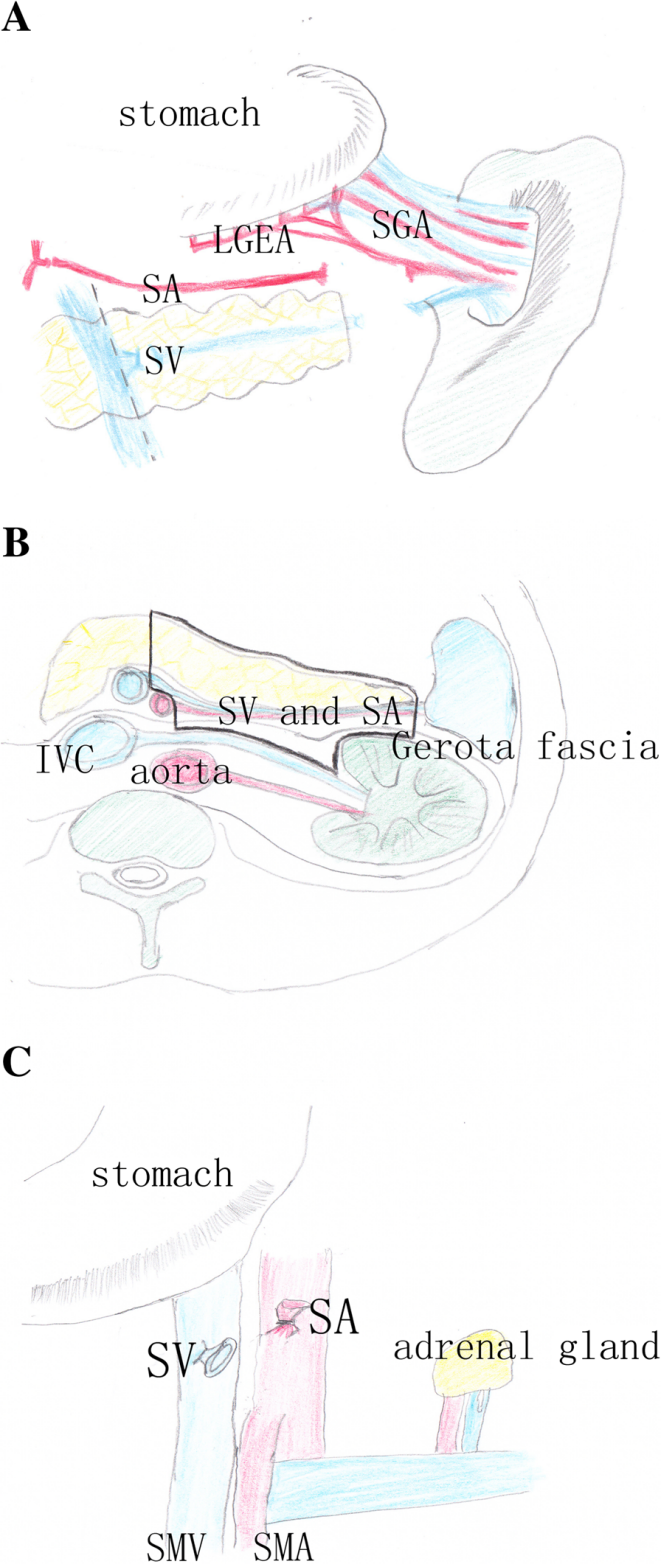
The diagram of en bloc resection range. **a** After dissecting the spleen artery and vein, the blood flow of the spleen was compensated by the left gastro-epiploic vessels and short gastric vessels. **b** The Gerota fascia, spleen artery and vein, and distal pancreas were removed with the en bloc technique. **c** The posterior peritoneal structures were shown after the specimen was removed

After this procedure, the common hepatic artery, left gastric artery, celiac trunk, SMA, left adrenal gland and its vessels, left kidney, and left renal vein are exposed. The en bloc distal pancreatectomy is finished (Figs. 2 and 3). At last, we closed the pancreatic duct, dislodged the specimen, and left a drainage tube at the surgical region.

### Data collection and statistical analysis

To analyze the complications after surgery, we adopt the Clavien-Dindo classification method [11]. To analyze the pancreatic fistula, we adopt the International Study Group of Pancreatic Fistula classification method [12]. The definition of spleen infarction was that the regional or total spleen is not enhanced by contrast-enhanced CT. The criteria of discharge were that the complications of the patient were stabilized, no analgesia was taken, and the patient could accomplish daily activity. The follow-up items after discharge included routine test, tumor markers (CEA, CA199), contrast-enhanced CT, or MRI according to the NCCN guideline [13].

#### Statistical analysis

Categorical variables were demonstrated as quantity or percentage. To compare between groups, we use Fisher’s test or chi-square. Successive variables were demonstrated as mean ± SD. To compare them, we use *t* test. Survival duration started from the date of the surgery. Recurrence-free survival curve was depicted by the Kaplan–Meier method. When we evaluated the recurrence-free survival time, we included the recurrence-free lethal cases. SPSS (version 19.0.2; SAS Institute Inc., Cary, NC) software was used in the statistical analysis.

## Results

Among the 23 pancreatic cancer patients who underwent LDP, 12 were males and 11 were females. Average age was 65 ± 11.4 years. Average BMI was 25.9 ± 4.4 kg/m^2^. Anesthesia risk score (ASA score) was 2.2. No patients underwent neo-chemotherapy before surgery. The information during the surgery was depicted in Table 2. There are 17 spleen-preserving LDP cases and six spleen-resecting LDP cases. Average surgery time was 203 ± 54 min. Average blood loss was 208 ± 264 ml. One case converted to ODP because of severe adhesion between the greater omentum, transverse colon, and intestine. There was no statistical significance in surgery time between the spleen-preserving LDP group and the spleen-resecting LDP group (198 ± 59 vs 223 ± 29 min; *P* = 0.45). There was no statistical significance in blood loss too (184 ± 65 vs 275 ± 109 ml; *P* = 0.49).

**Table 2:** The information during the surgery

Number of cases	
En bloc spleen-preserving LDP	17 (74 %)
En bloc spleen-resecting LDP	6 (26 %)
Surgical time	203 ± 54 min
Blood loss	208 ± 264 ml
Blood transfusion	0
Transfer to ODP	1 (4 %)

The complication information was showed in Table 3. The overall incidence of complication is 48 % (*n* = 11). The incidence of pancreatic fistula was 39 % (*n* = 9). There was no statistical significance in the complication rate between the two groups (48 %, *n* = 8 vs 50 %, *n* = 3; *P* = 0.90). All the patients were discharged within 30 days. There were four cases of grade IIIa complications. Two cases underwent percutaneous catheter drainage under the ultrasound scan. Two cases underwent ERCP to identify the pancreatic fistula. Three cases were diagnosed with spleen infarction. Twelve patients were not diagnosed with spleen infarction. Two patients did not take CT examination after surgery.

**Table 3:** The information after surgery

Clavien-Dindo complication degree	
Grade I or II	7 (30 %)
Grade III or IV	4 (17 %)
Pancreatic fistula degree	
Overall	9 (39 %)
Grade A	5 (22 %)
Grade B	3 (13 %)
Grade C	1 (4 %)
Operation method	
En bloc spleen-resecting LDP	(*n* = 17) 6 (35 %)
En bloc spleen-preserving LDP	(*n* = 6) 3 (50 %)
Pancreas partition method	
Harmonic scalpel (*n* = 20)	7 (35 %)
Ligasure (*n* = 3)	1 (33 %)
Spleen infarction	3 (18 %)
Mortality rate	0
Length during stay (days)	17 ± 8

All the patients who suffered from spleen infarction underwent conservative treatment, and all were cured without splenectomy. The average length of stay (LOS) was 17 ± 8 days. There was no statistical significance between the spleen-preserving and spleen-resecting LDP groups (18 ± 8 vs 15 ± 7 days; *P* = 0.36). The average dimension of the tumor was 32 ± 12 mm. The average number of lymph nodes that the specimen contained was 19.8 ± 9.3. There were 14 patients whose positive lymph node number is ≥1. The pathology results were 18 cases of ductal adenocarcinoma, four cases of intraductal papillary mucinous adenocarcinoma, and one case of mucinous adenocarcinoma. The surgical margins of all the patients were negative. The final pathology results of the patients were as follows: two cases of IA stage (8.5 %), five cases of IB stage (22 %), two cases of IIA stage (8.5 %), 13 cases of IIB stage (57 %), and one case of III stage (4 %) (Table 4).

**Table 4:** Pathology outcomes

Variables	Value, *n* (%) or mean ± SD
Histologic factors	
Tumor size (mm)	32 ± 12 (range, 10–60)
Final histologic diagnosis	
Ductal adenocarcinoma	18 (79 %)
Adenocarcinoma associated with IPMC	4 (17 %)
Mucinous cystadenocarcinoma	1 (4 %)
Differentiation	
Well	11 (48 %)
Others	12 (52 %)
Harvested LNs	19.8 ± 9.3 (range, 5–40)
Metastatic LNs	2.0 ± 4.1 (range, 0–20)
Surgical margin positive	0
Stage of tumor (UICC classification)	
pT1	3 (13 %)
pT2	8 (35 %)
pT3	11 (48 %)
pT4	1 (4 %)
pN0	9 (39 %)
pN1	14 (61 %)
p stage	
IA	2 (8.5 %)
IB	5 (22 %)
IIA	2 (8.5 %)
IIB	13 (57 %)
III	1 (4 %)

*IPMC* intraductal papillary mucinous carcinoma, *LN* lymph node, *UICC* Union for International Cancer Control classification, 7th edition

### Long-term prognosis

**Fig. 4:**
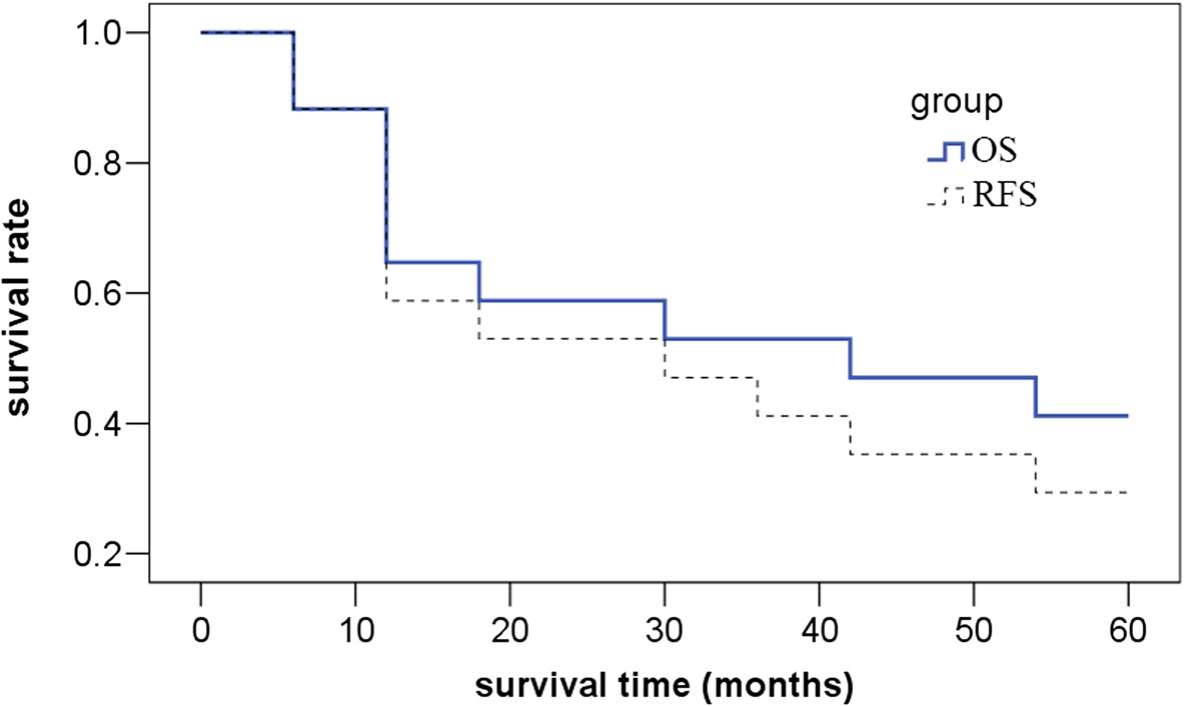
The overall survival (OS) and recurrence-free survival (RFS) curve. The observation period was 5 years after surgery. The average survival duration was 19 months. The survival rates of 1, 3, and 5 years after surgery were 64.7, 52.9, and 47.1 %, respectively. Six patients suffered from local recurrence, one suffered from local recurrence combined with liver metastasis, and two suffered from retroperitoneal recurrence. The average time to recurrence was 14 months. The recurrence-free survival rates of 1, 3, and 5 years after surgery were 58.8, 47.1, and 35.3 %, respectively

The observation period was 5 years after surgery. The average survival duration was 19 months. The survival rates of 1, 3, and 5 years after surgery were 64.7, 52.9, and 41.2 %, respectively. Six patients suffered from local recurrence, one suffered from local recurrence combined with liver metastasis, and two suffered from retroperitoneal recurrence. The average time to recurrence was 14 months. The recurrence-free survival rates of 1, 3, and 5 years after surgery were 64.7, 47.1, and 35.3 %, respectively (Fig. 4).

## Discussion

In recent studies [14, 15], the efficacy of radical en bloc LDP is satisfied for treatment of pancreatic cancer in long-term survival (5-year overall survival was 33 %). It is acceptable for treating pancreatic cancer. The data of our research was from a single center. The concept of en bloc LDP was based on R0 resection and adequate lymphadenectomy and ganglion resection.

Why do we preserve spleen? Adverse consequences have been observed after splenectomy. These include a greater likelihood of postoperative abscesses [16–18] and, most notably, a long-term risk of serious post-splenectomy sepsis. The risk of overwhelming post-splenectomy infection (OPSI) has been estimated to be 1 per 400–500 patient years and fatal OPSI to be 1 per 800–1000 patient years [19, 20]. While this risk is greatest in childhood, it persists to a lesser degree throughout life [21]. In addition, it increases the risk of later myocardial infarction, diabetes, and even cancer [22, 23].

How many types of spleen-preserving LDP surgery are in the world? There are mainly two types. One is invented by Kimura et al. [24] who performed spleen-preserving distal pancreatectomy with conservation of the splenic artery and vein. The advantage of this procedure was the low occurrence rate of spleen infarction. The disadvantage was isolating spleen vessels from the Toldt fascia which did not conform to the en bloc standards; in other words, this procedure is difficult. The other procedure was invented by Warshaw [25] who performed spleen-preserving distal pancreatectomy with resection of the splenic artery and vein. The blood flow of the spleen after surgery was compensated by the left gastro-epiploic vessels and short gastric vessels. Our procedure is similar with Warshaw’s procedure except for more extensive retroperitoneal resection, lymphadenectomy, and celiac ganglion resection.

Our study compared with recent studies (Table 5) shows that the number of lymph nodes obtained by LDP was similar with that by ODP (19.8 vs 15.5). One hundred percent R0 rate was our most satisfying outcome. The average dimension of the tumor was similar with that in ODP (32 vs 33.7 mm) too.

**Table 5:** The prognosis reported by previous studies about ODP

Source	Year	No. of cases	Median survival (months)	5-year survival (%)	Mean no. of harvested lymph nodes	Mean tumor size (mm)	Mean blood loss (ml)
Shimada et al. [6]	2006	88	22	19	NS	NS	NS
Kooby et al. [7]	2010	189	16	NS	13	25	790
Yamamoto et al. [8]	2010	73	NS	30	NS	33	707
Redmond et al. [9]	2010	94	16	NS	NS	43	NS
Mitchem et al. [10]	2012	47	26	36	18	34	744

NS: not stated

The decrease of blood loss during surgery compared with ODP (208 vs 747 ml) was mainly due to amplification of the view and precise manipulation under the laparoscope as well as the increased abdominal pressure.

The en bloc LDP technique emphasizes resecting the Gerota fascia, Toldt fascia, and spleen vessels and distal pancreas as a whole to ensure the negative surgical margin. In contrast, in the standard ODP, it did not need to remove the Gerota fascia and Toldt fascia routinely. It is a controversial topic if we should preserve the spleen. Some study [26] showed that the incidence rate of spleen infarction was 11 to 29 %. Till now, there has not been any guideline support that LDP should be combined with splenectomy.

Distal pancreatic cancers sometimes invade to the lymph nodes and ganglion around the superior mesenteric artery. But, totally dissecting the nerve plexus around the SMA often results in intractable diarrhea and vomiting. So, we preserved part of the nerve plexus at the right side of the superior mesenteric artery while dissecting lymph nodes and ganglions as previously reported [27].

Kim et al. [26] reported that there was a low incidence rate of lymphatic metastasis at the hilus lienis. Preserving the spleen can avoid the decrease of immuno-ability and improve the oncological prognosis. All these reports [16–23] prompted us to preserve the spleen. Till now, there has not been any re-operate case because of hilus lienis lymphatic metastasis.

It was Mitchem et al. [10] who reported that the average LOS after ODP was 11 ± 7 days. Kooby et al. [7] reported that the LOS after LDP was 7 ± 3 days. Because of the different discharge criteria, LOS is difficult to compare. The main reason for prolonged LOS is complications (47 %), such as pancreatic fistula (39 %). The optimized operative technique can reduce the incidence of complication. But, these techniques should also be summarized by a large number of cases of study.

In our study, the prognosis of en bloc spleen-preserving LDP was similar with the previous reports [8, 10, 28, 29]. In these reports, the average overall survival (OS) was 13 to 26 months, and the 5-year survival rate was 19–36 %. Till now, based on the reports we searched, it is the first study about the long-term prognosis of standard en bloc spleen-preserving LDP. The limitations of our research were the character of the retrospective study and the only three types of pathology results. Randomized controlled trials, a large number of cases, and long-term trials can avoid these disadvantages. But because the incidence rate of distal pancreatic cancer is low, the study is hard to carry out.

Nevertheless, en bloc LDP preserving the spleen is safe and effective. The short-term and long-term prognoses are satisfied which supports this procedure as a treatment for distal pancreatic cancer.

## Conclusions

The En-bloc spleen-preserving LDP surgery has good short-term and long-term outcomes for pancreatic cancers.

## Abbreviations

DP: distal pancreatectomy
LDP: laparoscopic distal pancreatectomy
LOS: length of stay
ODP: open distal pancreatectomy
OS: overall survival
SMA: superior mesentery artery
SMV: superior mesentery vein
